# Early onset of pre-lethal effects of lotilaner (Credelio^®^) on *Amblyomma americanum* ticks on experimentally infested dogs

**DOI:** 10.1186/s13071-021-04817-z

**Published:** 2021-06-13

**Authors:** Martha J. Wenger, Todd M. Kollasch, Madeline C. Burke, Livvy Jones, Casey Locklear, Makaela Hedberg, Lauren Miller, Sydnie Reeves, Debra Ritchie, Anthony J. Rumschlag, William G. Ryan, Vicki Smith, Cameron Sutherland, Kathryn E. Reif, Brian H. Herrin

**Affiliations:** 1 Department of Diagnostic Medicine/Pathobiology, College of Veterinary Medicine, Kansas State University, Manhattan, KS USA; 2 Elanco Animal Health Inc, 2500 Innovation Way, Greenfield, IN USA; 3 Ellen Jones, Manhattan, KS USA; 4 Ryan Mitchell Associates LLC, 16 Stoneleigh Park, Westfield, NJ USA

**Keywords:** Acaricide, *Amblyomma americanum*, Canine, Credelio, EthoVision XT, Isoxazoline, Lone star tick, Lotilaner, Motility

## Abstract

**Background:**

The speed with which acaricides paralyze and kill ticks is relevant to impeding pathogen transmission. The objective of this study was to assess early-onset lotilaner effects on the motility and weights of *Amblyomma americanum* ticks collected from treated dogs.

**Methods:**

Twelve healthy dogs were randomized between two groups to receive either lotilaner (Credelio^®^) on Day 0 or to be sham treated. On Day 7, 25 male and 25 female *A. americanum* were placed under bandages, two on each flank of each dog. After 30 or 45 min, all unattached ticks were removed and *T* = 0 was set. At *T* = 2, 4, 8 and 24 h post attachment, 5 attached ticks removed from each bandage on each dog were weighed, assessed by blinded observers for righting ability and movement recorded.

**Results:**

After the infestation period significantly fewer treated than control dogs had 20 ticks attached (50.0% versus 91.7%, *P* = 0.0015). At 24 h post attachment, mean weights of ticks from treated dogs (males 1.69 mg; females 2.72) were significantly less than ticks from controls (males 2.66 mg; females 4.67) (*P*_male_ = 0.0002; *P*_female_ < 0.0001). Mean tick weights from the treated group were significantly lower at 24 h than at earlier time points (*P*_male_ < 0.0307; *P*_female_ = 0.0021). At 4 and 8 h, significantly fewer ticks from treated (14.3%, 0.0%, respectively) than from control dogs could right (73.3%, 70.0%) (*P*_4h_ < 0.0001; *P*_8h_ = 0.0024) (at 24 h, all ticks from treated dogs were dead), and distance moved was significantly less at all time points (*P*_2h_ = 0.0413; *P*_4h_, *P*_8h_ < 0.0001). Mean and maximum velocity of ticks from treated dogs were significantly lower, relative to controls, at 4 and 8 h (*P* ≤ 0.0001). Within the treated group, collected ticks had significantly lower mean and maximum velocities at 4 and 8 h compared to 2 h (*P*_mean_ < 0.0042; *P*_max_ < 0.0194).

**Conclusion:**

The observed changes indicate that lotilaner may disrupt tick attachment. In ticks that attached, a progressive impairment of neuromuscular processes began within 2 h. Those irreversible changes could substantially reduce the risk of pathogen transmission from tick to host.

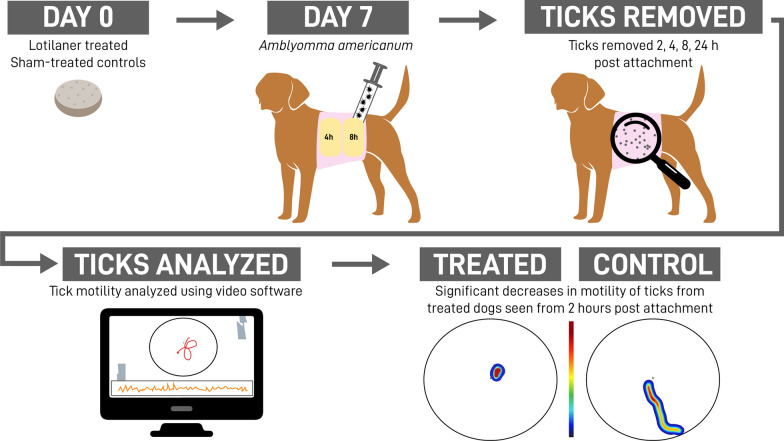

## Background

Discovery of the isoxazoline class of compounds provided a breakthrough of long-lasting, systemically acting options for the control of ticks on dogs. All marketed members of the class have demonstrated their tick-killing activity in studies conducted according to the guidelines of the World Association for the Advancement of Veterinary Parasitology [1]. According to those guidelines, efficacy of a tested product against ticks should be based on a ≥ 90% reduction in live tick infestation numbers, relative to untreated controls, within 48 to 72 h following treatment and following post-treatment challenge. While this is the current standard for product evaluation, the protocol creates several information gaps for the isoxazoline class. First, labeling these products as effective within 48–72 h leaves questions as to the ability of the isoxazoline class to prevent the transmission of tick-borne pathogens during that period. Those pathogens include *Borrelia* spp. and *Babesia* spp. (transmission within 24–48 h of initial feeding), *Rickettsia rickettsii* (transmission within 5 to 20 h after attachment) and *Ehrlichia* spp. (transmission within 3 to 6 h of attachment) [2‒5]. Although several isoxazolines have shown the ability to block specific pathogens experimentally, including lotilaner preventing the transmission of *Babesia canis* to dogs from infected *Dermacentor reticulatus* ticks, veterinarians and consumers may still have concerns about that interim feeding period [6].

Additionally, the recommended assessment of whether a tick is alive is through observation of any movement after gentle touching with a probe, CO_2_ exposure or warming [1]. A rationale for that methodology is that partially fed ticks that survive an initial drug exposure from a treated host may detach and, in the absence of continued exposure to the acaricide, recover to find a second host to complete the feeding process. This may be relevant because an interrupted feeding pattern has been shown to result in substantially reduced transmission times of pathogens, including *Rickettsia* spp., *Babesia* spp. and *Borrelia* spp. [7]. However, in some cases, that rigorous methodology for confirming post-exposure tick death may underestimate the true efficacy of a tested product. For instance, in one study the efficacy of lotilaner against an experimental challenge of dogs with *Ixodes ricinus* was 69.8% at 4 h post treatment [8]. However, following a 24-h incubation of ticks taken from study dogs, the total reduction in numbers of live ticks from treated dogs, relative to untreated controls, was 97.2%. A study was therefore initiated with the objective of generating further insight into the post-exposure behavior of ticks attached to lotilaner-treated dogs.

Of the four major tick species infesting dogs in the USA (*Rhipicephalus sanguineus, Ixodes scapularis, Dermacentor variabilis, Amblyomma americanum*), the least sensitive to the class of isoxazolines is *A. americanum*. The relative insensitivity of this tick is evident from the shorter post-treatment efficacy duration of fluralaner compared with the other three species (8 weeks versus 12 weeks) [9, 10]; for afoxolaner and for the product that combines the isoxazoline sarolaner with moxidectin and pyrantel, efficacy against *A. americanum* was determined by tick counts taken at 72 h following post-treatment infestations, rather than at the 48-h interval used for other labeled ticks [11, 12]. While lotilaner has been demonstrated to be effective against *A. americanum* for the full post-dose label period (up to 30 days) based on 48-h counts, the genus is regarded as being less susceptible than other tick genera [13]. That *A. americanum* may be a vector of *Rickettsia* spp. and *Ehrlichia* spp. adds to its importance as a canine parasite. Thus, this study was designed to provide a rigorous test of the pre-lethal effects of lotilaner treatment against a tick that is dose-limiting for the isoxazolines.

## Methods

### Dogs and management

Twelve (four female, eight male) purpose-bred, clinically healthy Beagle dogs, uniquely identified by ear tattoo, weighing 9.5 to 14.7 kg and all approximately 5 years of age, were selected from the Kansas State University colony. The dogs had been acclimated to the climate-controlled (21.7–23.3 °C; humidity: 11–33%) facility prior to study enrollment and were pair-housed in two stainless-steel kennels, each 4 × 6 ft (total 48 ft²), except for the day of tick infestations when they were individually housed. Dogs were excluded if they had a history of previous exposure to ticks, treatment with any isoxazoline product within the 6 months prior to starting the study or treatment with any topically applied acaricide/insecticide or acaricidal/insecticidal collar within the previous 30 days. None of the study dogs had been treated with any other long-acting insecticide/acaricide within the 12 months prior to Day 0. Prior to the study, testing for circulating antibodies (IDEXX SNAP 4DxPlus assay, and IFA) had been negative to *Borrelia* spp., *Ehrlichia* spp. and *Anaplasma* spp. Water was provided ad libitum in bowls, and a commercial ration was provided daily for each dog according to the manufacturer’s recommendation for an adequate diet. On the morning of administration of the study treatment (Day 0), feed was withheld from all dogs until within a half hour before the scheduled time of treatment. Health observations were completed daily by trained observers.

### Randomization and treatment

The 12 dogs were weighed and ranked by bodyweight, and, within sex, consecutive dogs were paired. Within blocks, each dog was randomly allocated to one of two treatment groups. On Day 0 dogs in Group 1 received a single treatment with a lotilaner chewable tablet (Credelio^®^, Elanco, Greenfield, IN, USA) at a minimum dose rate of 20 mg/kg (actual doses rates ranged from 22.7 to 39.5 mg/kg). The product was administered according to label, including the feeding of at least 1/3 of the dog’s daily ration within a half hour of treatment. Dogs in Group 2 were sham treated by mock-dosing and did not receive any acaricidal treatment. Immediately following dosing and return to their cages, dogs were observed to ensure that treatment was retained and to monitor for any adverse events.

### Tick infestations and removal

Adult *Amblyomma americanum* were purchased from a central tick rearing facility (EctoServices; Henderson, NC) and maintained in humidity chambers for a minimum of 4 weeks after molt from the nymphal stage. On Day 6 groups of 25 male and 25 female ticks were placed into a modified 3-ml syringe with moist dental wicks blocking both ends. The sorted ticks were placed back in the humidity chambers until infestation the following day. On the same day, the left and right thoracic and abdominal flanks of each dog were clipped with 40 blade (0.25 mm) clippers.

On Day 7 dogs were sedated with dexmedetomidine (Dexdomitor^®^, Zoetis, USA; 500 mcg/m^2^) and maintained in sternal recumbency throughout the infestation process. Four large patch bandages (2.875 × 4.0 in.; Band-Aid Brand Water Block large adhesive pad) were placed, one onto each clipped area. Each bandage was labeled with its respective dog number and removal time point (2, 4, 8, 24 representing the hours when ticks would be collected following attachment) and left open on one edge. One syringe containing 25 male and 25 female *A. americanum* was inserted into the pocket formed by each patch bandage, ticks were deposited, and the bandage was then securely sealed to the skin using firm pressure along the edges. This process was repeated for all four patches so that each dog was challenged with 200 ticks (25 males and 25 females under each of 4 bandages). Each time point patch was placed on the same site on the dogs (e.g. 24-h patch was the cranial patch on the left side of each dog).

After 30 min the bandages were removed. If at least 20 ticks had attached under a bandage, that was considered attachment time point zero (*T*0); all loose ticks were removed and fresh bandages applied. If fewer than 20 ticks were attached the loose ticks were replaced for another 15 min, after which all loose ticks were removed, fresh bandages were applied, and that was considered *T*0 for that dog. Thus, for any given dog, *T*0 could be either 30 or 45 min post-initial infestation. The 2-, 4-, 8- and 24-h time points for removing ticks were based on *T*0 (i.e. time of observed attachment), rather than on time of initial infestation. The dogs were then fitted with a 10″ tubular stockinette secured at the neck and waist with elastic adhesive tape (Elastikon^®^). A secondary stockinette and Elastikon layer were applied as well as an Elizabethan collar to prevent damage to the infestation chambers. After tick infestation, approximately 1 h post-sedation, the dogs were given a reversal dose of atipamezole (Antisedan^®^, Zoetis, USA; 5000 mcg/m^2^) per label, and monitored until upright and alert.

Using forceps and taking care to ensure ticks were not damaged, all attached ticks under one bandage were removed from each dog at *T* = 2, 4, 8 and 24 h, and the stockinette was resecured to protect the remaining bandages. After removal from each dog, ticks that had been attached were stored in small plastic containers until they could be further processed, all within 30 min of being removed from their hosts. For each time point from each dog, five ticks were weighed and video recorded for movement analysis. Any ticks removed from a study animal but not needed for the motility experiments were placed into small plastic containers in a humidity chamber. The live/dead status of those ticks was assessed after 24 h incubation [8].

All persons involved in infestations and tick collections were blinded to treatment groups. Infestation teams changed between treatment groups throughout the course of the study.

### Tick viability assessments

For motility analysis, each of five ticks taken for each time point from each dog was placed dorsal surface down onto a white paper arena (17.8 cm diameter) and allowed to right itself (righting is the ability for a tick placed on its dorsal surface to flip over and begin directional movement). The time from initial leg movement to upright was recorded, and ticks taking longer than 30 s were recorded as “did not right” [14]. After righting, the tick was placed upright into the center of the arena and given a CO_2_ stimulus by a breath from the blinded investigator. Subsequent movements were recorded until the tick left the arena or for 1 min, at which time there was adequate video to assess the tick’s movement. Digital recordings of each tick’s movements were saved onto a Secure Digital card, transferred to a university-owned shared server and uploaded into EthoVision XT software.

Once in the video software, a new arena calibration was created for each dog time point, resulting in detection and arena settings appropriate for the five ticks collected from a specific dog and time point. Total distance, maximum velocity, mean velocity and total video duration parameters were selected in the data collection settings. Mean velocity is calculated in the software as an average of the velocity of the tick while it is moving, not factoring in periods of time in which the tick stops moving. Trial control settings were left as the automatic settings in the software. Maximum and minimum detection settings were changed to 600 and 40, respectively, but were manually altered to a smaller range for videos in which the detection settings could not follow the tick. Each video was monitored during data capture to ensure the detection settings were able to detect the tick throughout the entirety of the recording and only tracked tick movement (the software could not distinguish between purposeful tick movement and extraneous movement such as forceps entering the arena). The data were then exported to Excel for statistical analysis. After filming activity levels, the ticks were weighed on a calibrated balance.

### Statistical analysis

The experimental unit was the attachment time point. The Kruskal-Wallis *H* test was used when comparing the means of multiple time points within a treatment group, with post hoc analysis to compare the specific time points against each other. The Mann-Whitney *U* test was used to compare, within sex, the mean weights of male and female ticks in each group at the 2-h post attachment time point to the mean weights at the 4-, 8- and 24-h time points. The Mann-Whitney *U* test was also used to compare between-group differences at each post-attachment time point for the mean duration of video recordings following placement of each tick in the center of the 17.8 cm arena, the mean distance moved by ticks, and mean velocity and maximum velocity of ticks collected from dogs in each group. Total numbers of attached ticks in each group and the mean time for tick righting at each post-attachment time point were compared, within and between groups, using the *χ*^2^ test. Statistical analysis was carried out using BioStat^®^ (AnalystSoft). Testing was two-sided at the significance level, *α* = 0.05.

## Results

**Fig. 1:**
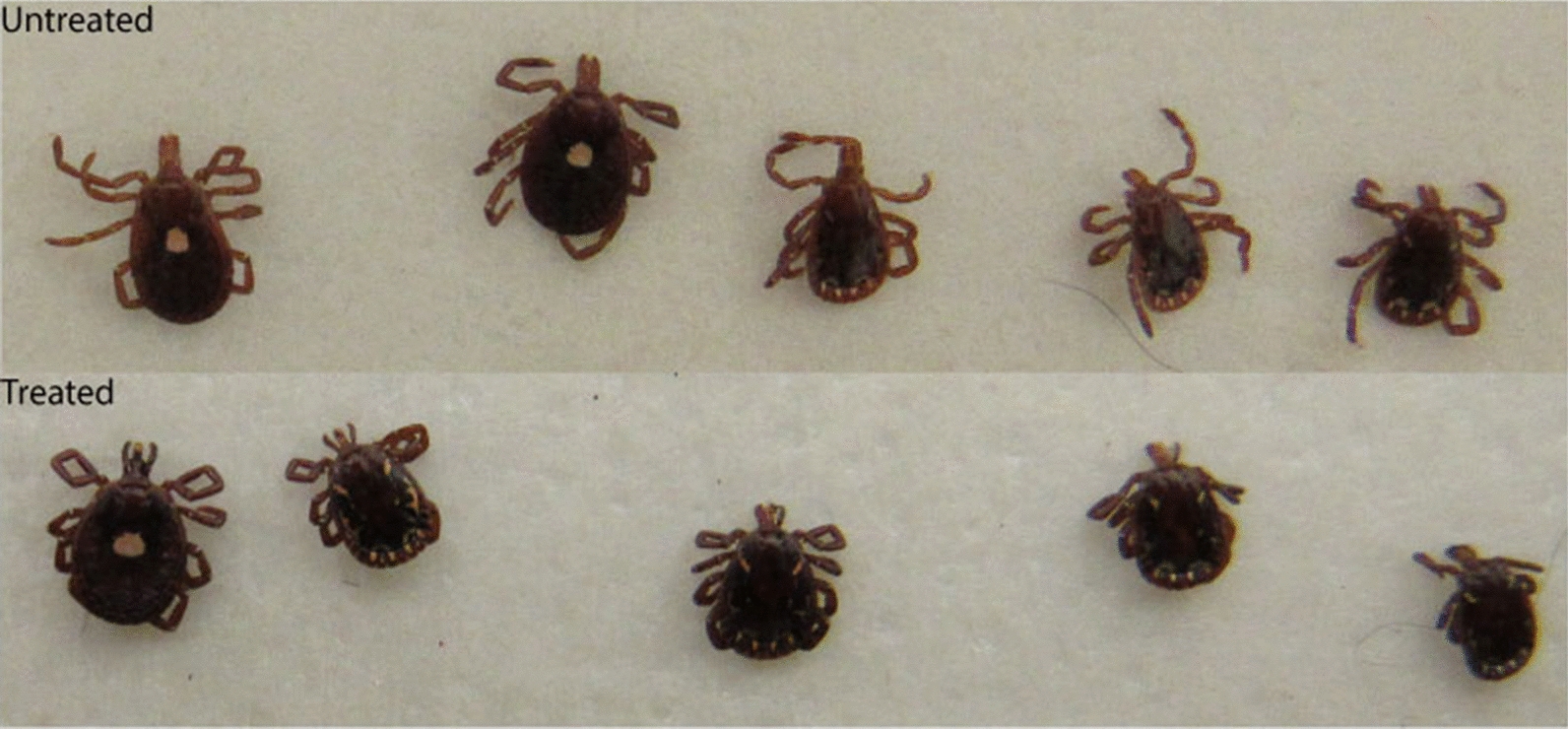
At 2 h post attachment following 24 h incubation: live *Amblyomma americanum* removed from sham-treated control dogs (top); dead *A. americanum* removed from lotilaner-treated dogs (bottom). Note between-group differences in legs and mouthparts

Ticks established attachment on all study dogs within the 45-min infestation period. Across all time points significantly more control dogs had at least 20 ticks attached under each bandage (22/24; 91.7%) than did treated dogs (12/24; 50.0%) (*χ*^2^ = 10.08; *P* = 0.0015). After removing the required number of attached ticks for study analyses, 7 extra ticks were collected from treated dogs at the 2-h time point, none at any other time point. From control dogs 11, 24, 37 and 17 extra ticks were removed at the 2-, 4-, 8- and 24-h time points, respectively. After incubation in a humidity chamber for 24 h, the 7 ticks removed from lotilaner-treated dogs at the 2-h post-attachment time point had died, while all the control ticks were alive (Fig. 1).

For ticks from control dogs (118 ticks), there were no significant changes in weights in either males or females across the four post-attachment time points (*H*_male_ = 7.0, *P*_male_ = 0.0727; *H*_female_ = 4.17, *P*_female_ = 0.2445). At the 24-h post-attachment time point, the mean weights of male and female ticks from treated dogs (116 ticks) were significantly less than at any earlier time point (Z_male_ = 2.16, *P*_male_ = 0.0307; Z_female_ = 3.07, *P*_female_ = 0.0021), and mean weights of ticks collected from these dogs were significantly lower than those from control dogs (males, *Z* = 3.71; *P* = 0.0002; females, *Z* = 3.97; *P* < 0.0001) (Table 1). There were no significant differences in tick weights between the 2-, 4- or 8-h time points within either group.

**Table 1:** Mean weights (mg) (95% confidence interval) of *Amblyomma americanum* ticks removed from lotilaner-treated and from sham-treated control dogs at 2, 4, 8 and 24 h post attachment

Time post attachment (h)	Males				Females			
	Treated	Control	*Z*-value	*P*-value	Treated	Control	*Z*-value	*P*-value
2	2.45 (2.18‒2.71)	2.29 (2.13‒2.45)	1.00	0.3222	4.41 (3.96‒4.86)	4.30 (3.43‒5.17)	0.04	0.9650
4	2.32 (2.10‒2.54)	2.64 (2.35‒2.92)	1.55	0.1214	3.92 (3.39‒4.44)	4.08 (3.78‒4.39)	0.54	0.5865
8	2.17 (1.86‒2.48)	2.44 (2.22‒2.67)	1.02	0.3062	3.92 (3.43‒4.42)	4.44 (2.18‒2.71)	1.47	0.1419
24	1.69 (1.47‒1.92)	2.66 (2.32‒3.01)	3.71	0.0002	2.72 (2.29‒3.16)	4.67 (4.13‒5.22)	3.97	< 0.0001

**Fig. 2:**
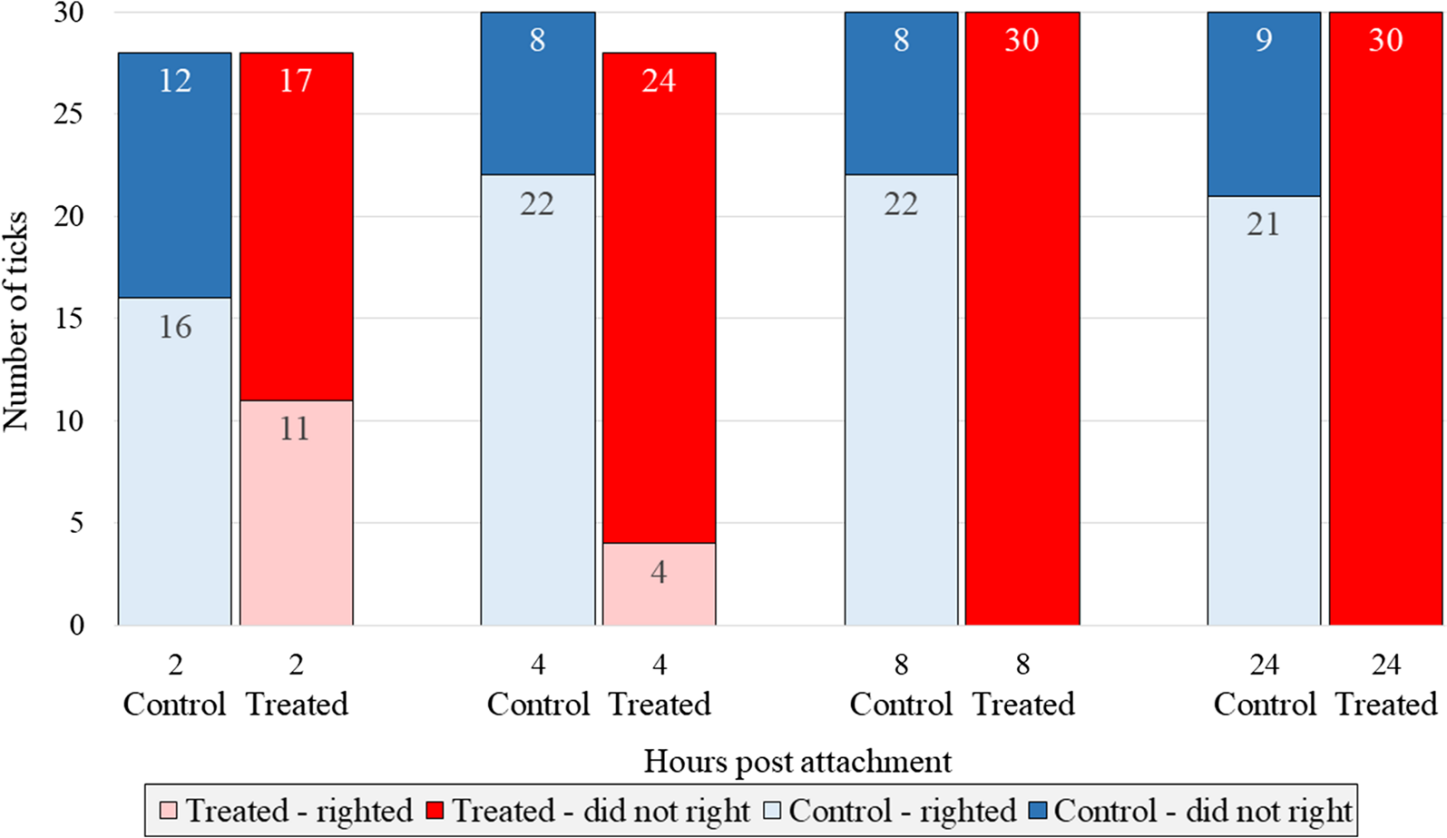
Number of *Amblyomma americanum* ticks taken from lotilaner-treated and sham-treated control dogs that were able to right within 30 s of being placed upside down (no ticks taken from treated dogs were able to right at 8- and 24-h time points post attachment, i.e. value = 0)

After being placed dorsal surface down onto a white paper arena, each tick was allowed a maximum of 30 s to right (flip over). At 4 h post attachment, significantly more ticks from control dogs 22/30 (73.3%) could right compared to only 4/28 (14.3%) from treated dogs (*χ*^2^ = 20.42; *P* < 0.0001) (Fig. 2). At 8 and 24 h post attachment, none of the ticks from treated dogs were able to right (0/30 at 8 and 24 h; both 0.0%), whereas 22/30 (73.3%) of the 8-h and 21/30 (70.0%) 24-h time point ticks from control dogs were able to right (*χ*^2^_8h_ = 34.74; *P*_8h_ < 0.0001; *χ*^2^_24h_ = 32.31; *P*_24h_ < 0.0001).

### Tick motility

**Fig. 3:**
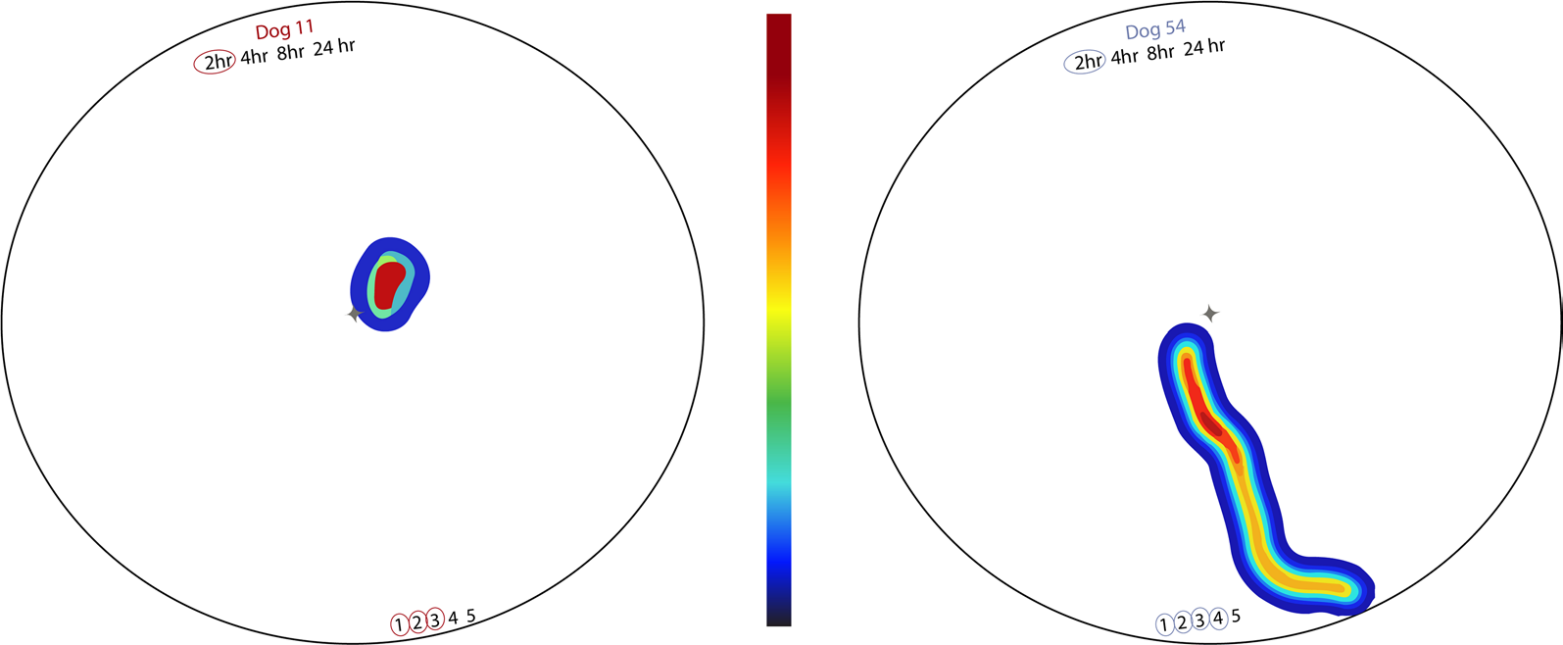
Example of heat maps created using the video software. Left: heat map of *Amblyomma americanum* tick from lotilaner-treated dog; Right: heat map of tick from sham-treated control dog. Darker blue represents less time spent in the arena, darker red more time

The video analysis was started when a tick first started moving in the arena and was stopped when it left the arena or after 1 min (Fig. 3). The video software could appropriately analyze the recordings from 67 ticks collected from control dogs, and 61 from treated dogs. All the 24-h time point videos of ticks from both study groups were on a secondary memory card that was inadvertently cleared before backing up. At this point, all ticks from treated dogs were dead.

Significant between-group differences were observed in video duration at the 4- and 8-h post-attachment time points when the videos of ticks from treated dogs were significantly longer than those of ticks from control dogs (Table 2). For ticks from treated dogs a significant increase was found in mean video duration from 2 to 4 h post-attachment (*Z*_2v4_ = 3.07, *P*_2v4_ = 0.0022) and at 2 to 8 h post attachment (*Z*_2v8_ = 2.72, *P*_2v8_ = 0.0065). There were no significant between-time point differences for ticks taken from control dogs.

**Table 2:** Mean duration (s) of video recording (95% confidence interval) of *Amblyomma americanum* ticks removed from lotilaner-treated and from sham-treated control dogs

Time post attachment (h)	Treated time (s)	Control time (s)	Mann–Whitney *U* test (treated *vs* control)	
			*Z*-value	*P*-value
2	18.4 (14.7–22.7)	20.7 (15.0–26.4)	0.51	0.6099
4	31.6 (32.9–39.3)	16.9 (12.3–21.5)	3.24	0.0012
8	29.9 (23.7–36.2)	20.2 (13.5–27.0)	2.91	0.0036

Within-group comparison of multiple independent samples				
Kruskal–Wallis *H*-value	11.71	1.19		
*P*-value	0.0029*	0.5498		

^*^The 2-h post-attachment videos were significantly shorter than those at 4 and 8 h

At each measured post-attachment time point, significant differences were evident between the mean distance moved by ticks collected from sham-treated control dogs and ticks from lotilaner-treated dogs (Table 3). No significant differences were found between post-attachment time points in video durations of ticks from control dogs (*H* = 0.4101; *P* = 0.8146). With increasing time from attachment there was a significant reduction in mean distance moved by ticks from treated dogs between both the 4- and 8-h time points relative to the 2-h time point (*Z*_2v4_ = 1.98, *P*_2v4_ = 0.0482; *Z*_2v8_ = 3.00, *P*_2v8_ = 0.0027) but not between the 4- and 8-h time points (*Z*_4v8_ = 1.18, *P*_4v8_ = 0.2381).

**Table 3:** Mean distance (cm) (95% confidence interval) moved by *Amblyomma americanum* ticks collected from lotilaner-treated and from sham-treated control dogs during 1-min observation or until moving beyond the video perimeter

Time post attachment (h)	Treated distance (cm)	Control distance (cm)	*Z*-value	*P*-value
2	7.10 (4.07–10.13)	10.25 (7.66–12.84)	2.04	0.0413
4	3.61 (1.72–5.51)	11.01 (7.87–14.15)	3.92	< 0.0001
8	1.74 (0.25–3.23)	10.70 (7.95–13.46)	4.49	< 0.0001

At 4 h and 8 h post attachment, but not at 2 h, ticks collected from treated dogs had significantly lower mean and maximum velocity than those from control dogs (Table 4). There were no significant between-time point differences in mean or maximum velocity of ticks from control dogs (*H*_mean_ = 2.31, *P*_mean_ = 0.3155; *H*_max_ = 0.84, *P*_max_ = 0.6582). Ticks from treated dogs had significantly higher mean and maximum velocities at the 2-h compared to the 4-h time point (*Z* = 2.34, *P* = 0.0194) and 8 h (*Z* = 3.74, *P* = 0.0002), but the 4- and 8-h time points did not differ significantly from each other (*Z* = 1.75, *P* = 0.0806).

**Table 4:** Mean and maximum velocity (cm/s) (95% confidence interval) of *Amblyomma americanum* ticks collected from lotilaner-treated dogs and from sham-treated control dogs

Time post attachment (h)	Treated	Control	*Z*-value	*P*-value
Mean velocity (cm/s)				
2	0.56 (0.33–0.79)	0.65 (0.49–0.82)	1.17	0.2435
4	0.17 (0.05–0.30)	0.82 (0.64–1.00)	4.76	< 0.0001
8	0.07 (0.00–0.16)	0.74 (0.58–0.91)	4.46	< 0.0001
Maximum velocity (cm/s)				
2	1.15 (0.88–1.42)	1.64 (1.15–2.12)	1.65	0.0985
4	0.70 (0.39–1.02)	1.54 (1.29–1.78)	3.81	0.0001
8	0.34 (0.17–0.51)	1.41 (1.17–1.64)	4.70	< 0.0001

## Discussion

Following oral administration to fed dogs, blood concentrations of lotilaner can be detected within 30 min and peak blood concentrations are achieved within 2 h [15, 16]. The terminal lotilaner half-life of 30.7 days and mean residence time of 45.3 days mean that acaricidal blood levels are sustained through at least 35 days following treatment [15]. Thus, the *A. americanum* challenge in this study, at 7 days post-treatment, was completed in the presence of acaricidal blood concentrations of lotilaner. The 7-day assessment was chosen to avoid the immediate post-treatment peak in lotilaner blood concentrations but does not allow definitive conclusion about the effects beyond 7 days post treatment. However, the sustained lotilaner blood levels and efficacy against *A. americanum* shown to remain at 100% through 30 days following treatment suggest that the effects observed in the study would continue beyond the study observation period [16].

### Tick attachment

Perhaps related to those concentrations there was a significant if incomplete reduction in tick attachment to treated dogs relative to control dogs. During the infestation day the personnel infesting each group of dogs changed, such that one team did not infest all treated or all control dogs. There were also no trends to suggest that conditions under any one patch were more tick-favorable than those under other patches, that ticks preferred the cranial or caudal patches or preferred the left or right side of the dog. The finding of significant between-group differences in the attachment rates was unexpected and raises many questions, as results of this nature where ticks are not able to attach and feed are typically associated with studies involving non-systemic, topically-applied acaricides, such as pyrethroids [17]. Such acaricides are known to disrupt ticks before the attachment and feeding process begins because of their repellant and anti-feeding properties [18]. In the study that we report, the reduced tick attachment rates on treated dogs suggests that lotilaner may have rapid onset effects that potentially disrupt the early probing and attachment of ticks even before the feeding process begins. This probing/seeking behavior has been observed by one of the authors of this paper (BHH, data unpublished). This phenomenon is supported by repeated observations of *A. americanum* preferentially feeding in the inguinal and axial regions of dogs and horses [19, 20]. In nature, these ticks may initially tack down to safely stay on a host until they can move to a preferential feeding site. As this is the first report of a systemic acaricide disrupting tick attachment, further controlled studies are needed to confirm this early effect of lotilaner and whether it is applicable to other isoxazoline compounds and to other tick species.

### Tick weight

The mean weight of ticks taken from control dogs did not increase significantly between 2 and 24 h post attachment. This is consistent with published literature on the early stage of tick feeding where imbibing blood and injecting saliva is approximately equivalent and overall blood intake is minimal [21, 22]. In contrast, at 24 h post attachment ticks collected from lotilaner-treated dogs had lower mean weights than those from control dogs and lower mean weights than at 2 h post-attachment. The observed weight loss is likely due to failure of a dying/dead tick’s cuticular water balancing activity and inability to replenish water loss through feeding. This would support the idea that in ticks exposed to treated dogs the muscular pumping mechanisms used to maintain water balance are shutting down during or soon after the process of attachment. Further analysis of tick salivary glands may clarify whether those effects include inhibition of the upregulation of the salivary proteins that regulate water balance and blood feeding.

### Motility assays

To our knowledge, this is the first study using EthoVision XT software to analyze tick movements post-exposure to acaricidal products. This software has been used to monitor attractant behaviors in ticks and insects and to document the responses to insecticidal products in a variety of insects [22‒26]. The software was able to accurately detect tick movement with sufficient accuracy to allow analysis of distance and velocity parameters but was inaccurate in assigning directional and angular movements. The duration of recordings of ticks from treated dogs was significantly longer than those from control dogs at the 4- and 8-h time points because ticks from control dogs generally moved more directly towards the CO_2_ stimulus, leaving the arena. In contrast, ticks from treated dogs showed less purposeful movement, wandering in circles or failing to move at all, resulting in videos that ran for a full 60 s without the tick leaving the arena. Rather than limiting or standardizing the videos to the fastest control tick leaving the arena, the videos were analyzed in their entirety to give the treated ticks full opportunity to move throughout the arena.

The average distance traveled by the control-dog ticks at any time point was approximately 10.5 cm, which corresponds to their direct movement out of the arena. The radius of the arena was 8.9 cm, and the majority of ticks from the sham-treated control dogs would respond to the CO_2_ stimulus by reorienting and moving directly toward the stimulus and out of the arena. The total distance traveled by ticks from treated dogs was significantly lower than that of control dogs at each time point. For ticks collected from treated dogs at the 2-h time point, many were able to move directionally toward the stimulus, while some appeared incapable of such movement, and the mean distance covered by these ticks was less than the radius of the arena. Thus, while ticks from the control dogs were able to directionally ambulate, any movement of those collected from treated dogs was generally non-directional and circular, and the treatment effect increased with the time that ticks had been attached. These early effects of lotilaner became increasingly obvious from 4 h post attachment when only 14% of ticks collected from treated dogs could right within 30 s compared with 73% of ticks taken from control dogs. At 8 and 24 h post attachment, no ticks from treated dogs could right, compared with successful righting by 73% and 70% of ticks from control dogs, respectively. The velocity parameters mirrored that of distance and righting, with significantly lower mean and maximum velocities in ticks from the treated dogs compared with ticks from control dogs at the 4- and 8-h time points.

Overall, these findings are indicative of a treatment effect interfering with tick motility at all assessed time points, beginning at the first observation 2 h post attachment. This early interruption of neuromuscular activity may play a key role in isoxazoline blocking of the transmission of tick-borne pathogens. An animated model of the feeding apparatus of *Ixodes ricinus* has highlighted ways in which tick feeding and salivation processes are regulated by neuromuscular activity [27]. First, the pharyngeal valve opens and the pharynx dilates to imbibe blood, then the valve closes and the pharynx contracts, pushing the bloodmeal into the midgut. Disruption of the muscles controlling these processes would result in an inability to imbibe or regurgitate blood, thereby preventing its movement through the tick’s digestive system. For many tick-borne pathogens the contents of the bloodmeal are key signals to proliferate or migrate to the salivary glands to facilitate transmission [28, 29]. A decrease in blood intake could reduce the signal needed to prepare these pathogens for transmission. Additionally, muscles attached to the salivarium are likely involved in the expulsion of saliva into the host, such that paralysis of these muscles via isoxazoline exposure would potentially further reduce the risk of pathogen transmission [27]. While our study did not directly measure neuromuscular potential or study the muscles involved in the feeding apparatus, isoxazoline compounds act by blocking insect and acarine GABA- and glutamate-gated chloride channels, providing a basic mechanism for disrupting the transmission of pathogens [30‒32]. Ticks exposed to pre-lethal doses of these compounds may remain attached to a host because of the physical hooks of the hypostome and chelicerae, but, at some point, become unable to feed at all.

Although the disruption of tick attachment in treated dogs was unexpected, it was not complete, and 7 extra attached ticks were removed from treated dogs at the 2-h time point. These ticks were then incubated along with 11 extra ticks collected from control dogs at this time point. All the ticks from the treated dogs died during the 24 h incubation while all incubated ticks from the control group survived, indicating that ticks were exposed to a fatal dose of lotilaner within 2 h of attachment to a treated dog, although those effects were not seen until later. Thus, the lethality of lotilaner may not be immediately evident in study observations, but it would appear that the effects on tick neuromusculature begin before or very soon after attachment is initiated and are irreversible. In the ticks from treated dogs, desiccation likely occurred because of an inability to produce hygroscopic saliva to maintain water balance compared to the untreated ticks [22]. A similar increase in lethality has already been reported for sarolaner’s ability, as a single entity product, to kill *I. scapularis* and *A. americanum* and lotilaner’s ability to kill *I. ricinus* after removal and incubation from treated hosts, indicating that once a tick is affected it would not be able to locate and attach to another host [8, 33]. This finding is highly relevant to situations of interrupted feeding, where a tick separates during feeding and subsequently attaches to a second host, a phenomenon that has been shown to lead to greatly accelerated pathogen transmission [34, 35]. The incubated ticks taken from treated dogs were not inspected until 24 h after removal and may have died well before that. Additional studies are required to determine more precisely the interval between tick exposure to a treated dog and tick death.

### Limitations

The infestation goal was for each dog to have at least 20 ticks attached under each bandage that would then be removed for analysis. Patches that had at least 20 ticks attached were recorded as such without documenting the exact number, precluding a comparison between control and treated dogs. We were only able to analyze the groups categorically as “20 ticks attached” or “Not.”

The low rate of attachment to the treated dogs limited the numbers of ticks available to be held overnight in humidity chambers. While the few ticks that were pulled from treated dogs at the 2-h time point all died during the 24-h incubation, the number was too low to allow a statistical comparison. Nonetheless, this is a promising track for future projects.

Finally, the open arena format for recording tick movements may have resulted in an underestimate of the difference in distances moved between ticks taken from treated and control dogs. Thus, the healthiest ticks moved directly towards the CO_2_ stimulus, quickly exiting the arena, creating a short video with a short distance traveled, so that the videos of ticks from the control dogs were of shorter duration than those collected from treated dogs. Had there been some form of barrier preventing a tick leaving the arena it seems likely that healthy ticks would continue their movements laterally and vertically to reach the source of the stimulus, resulting in longer videos and greater distance covered. However, the analysis of mean and maximum velocity of the ticks helps  to more accurately describe the true motility abilities of the two groups.

## Conclusion

The results indicate that lotilaner may partially inhibit tick attachment. For ticks that did attach to treated dogs, the absence of purposeful movement became increasingly evident with time post attachment, suggestive of a progressive impairment of neuromuscular processes, beginning within 2 h post attachment. Death of incubated ticks, even after only 2 h attachment, indicated this effect was fatal. As the feeding and salivation of ticks are also regulated by neuromuscular processes, this study provides an initial basis for the mechanism by which systemically acting isoxazolines can reduce the risk of pathogen transmission prior to tick death.

## Data Availability

Data from this study are available for review on reasonable request to Elanco Animal Health or Kansas State University.
